# Three dimensional magnetization structure of the Tofua Arc 12 seamount constrained by magnetization vector inversion

**DOI:** 10.1038/s41598-026-46834-x

**Published:** 2026-04-03

**Authors:** Soon Young Choi, Hyung Rae Kim, Young Tak Ko, Won -Hyuck Kim, Hyun -Ok Choi, Jong Dae Do, Chang Hwan Kim

**Affiliations:** 1 Dokdo Research Center, East Sea Research Institute, Korea Institute of Ocean Science and Technology (KIOST), Uljin, Republic of Korea; 2 Division of Science Education, Kangwon National University, Chuncheon, Republic of Korea; 3 Department of Geoenvironmental Sciences, Kongju National University, Gongju, Republic of Korea; 4 Ocean Georesources Research Center, Korea Institute of Ocean Science and Technology (KIOST), Busan, Republic of Korea; 5 OMEGAS Earth and Environmental and Space Convergence Science Education and Research Center, Chungnam National University, Daejeon, Republic of Korea; 6 East Sea Environment Research Center, East Sea Research Institute, Korea Institute of Ocean Science and Technology (KIOST), Uljin, Republic of Korea

**Keywords:** Tofua Arc 12, Lau Basin, Back-arc seamount, Magnetization vector inversion (MVI), Marine magnetics, Hydrothermal alteration, Natural hazards, Ocean sciences, Solid Earth sciences

## Abstract

The internal structure of submarine arc volcanoes remains poorly resolved, limiting our understanding of the interactions among magmatism, tectonics, and hydrothermal circulation. We investigated the Tofua Arc 12 (TA12) seamount in the Lau Basin using multibeam bathymetry and shipborne magnetic data processed through International Geomagnetic Reference Field (IGRF) correction, reduction to the pole (RTP), analytic signal analysis, and three-dimensional magnetization vector inversion (MVI). Magnetic anomaly maps show elevated amplitudes along the caldera rim and lower amplitudes within the depression. The full-vector MVI approach further resolves depth-dependent variations in both magnetization amplitude and direction, with enhanced values from ~ 3 km below sea level (bsl) upward concentrated along the caldera rim and beneath the depression, consistent with possible sill-like intrusions and subsequent cone-building episodes. In contrast, relatively low magnetization in parts of the depression and slump sectors is consistent with hydrothermal alteration and/or disaggregated materials. Independent comparison with published seismic profiles provides qualitative constraints on rim-bounding faults, mass-wasting surfaces, and a shallow basement interface near ~ 2 km bsl. We propose a conservative three-stage evolutionary model involving an initial caldera-forming eruption, later intrusions and cone growth, and fault-controlled fluid circulation, while explicitly acknowledging the resolution limits imposed by shipborne survey line spacing. These results demonstrate that integrating full three-dimensional MVI with complementary geophysical constraints can provide a reproducible framework for imaging submarine arc volcanoes and guiding future near-bottom surveys and resource assessments in back-arc basins.

**Supplementary Information:**

The online version contains supplementary material available at 10.1038/s41598-026-46834-x.

## Introduction

**Fig. 1:**
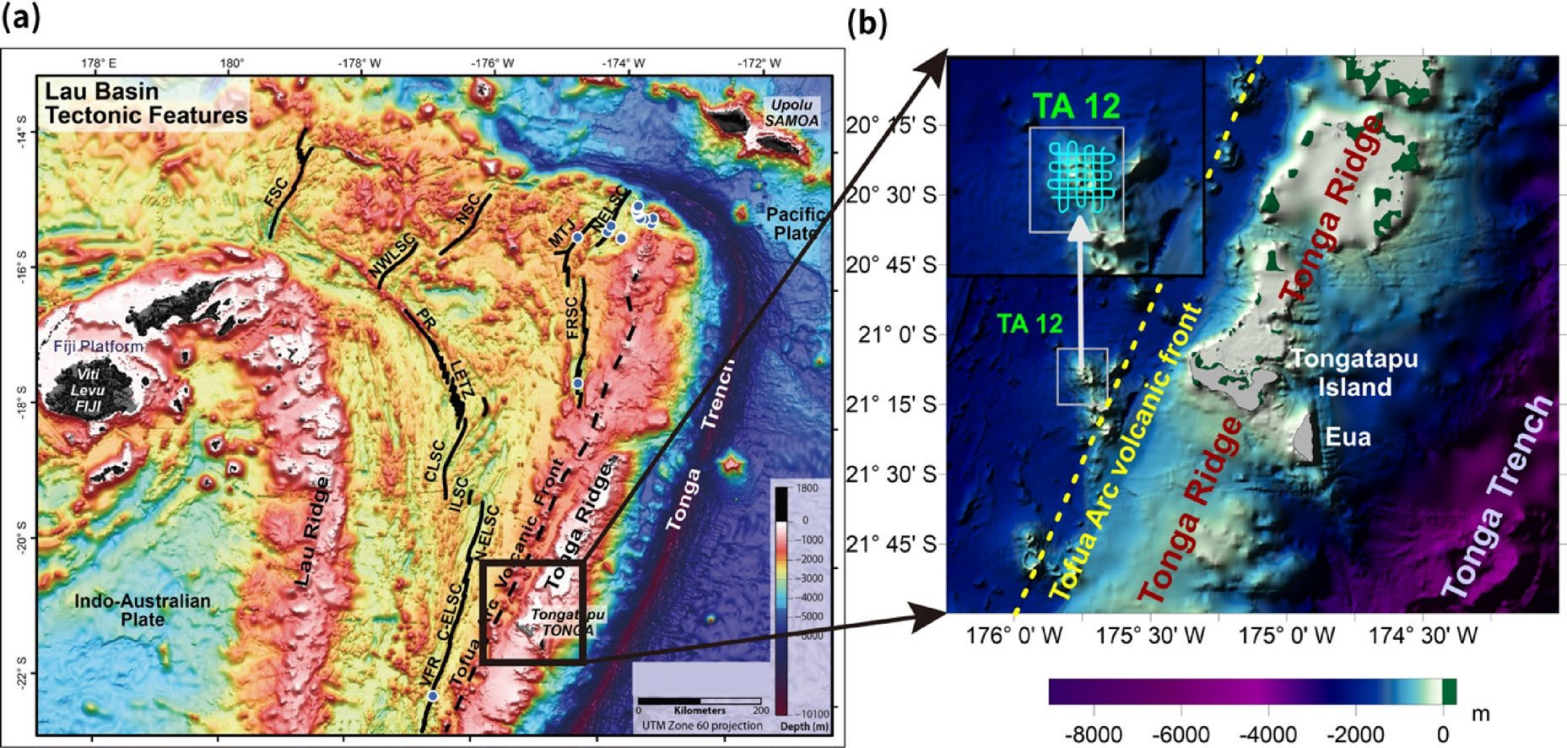
Regional bathymetry of the survey layout of Tonga Arc and TA12 seamount. Three-dimensional regional bathymetry of the Tonga Arc and Lau Basin region (depth in meters below sea level). The tectonic framework (inset, top right) is modified from ref. 2. The TA12 seamount, located along the volcanic front, is shown in the magnified inset (top left). Light blue lines indicate shipborne magnetometer survey tracks acquired during the 2009 KIOST expedition. Figure was generated using Surfer 13 (Golden Software, https://www.goldensoftware.com/) for data visualization, and Adobe Illustrator 2026 (Adobe, https://www.adobe.com/) for final figure composition. Panel (**a**) is reproduced from a previously published study. Panel (**b**) shows a magnified bathymetric view of the TA12 seamount and surrounding region, highlighting detailedseafl oor morphology and the survey area.

Seamounts in back-arc basin environments play a crucial role in understanding submarine volcanism, tectonic processes, and hydrothermal mineralization. The Lau Basin, located at the convergent boundary between the Pacific and Indo-Australian plates in the southwest Pacific Ocean, is bounded by the Lau and Tonga ridges. These ridges formed during east–west extension around the East and Central Lau Spreading Centers (ELSC and CLSC) following the counterclockwise rotation of the Fiji Platform at 3–5 Ma^1,2^ (Fig. 1). The Tonga Arc, located to the east of the Lau Basin, represents the active volcanic front of this back-arc system. Both the Lau Basin and the Tonga Arc are relatively young (< 10 Ma) and host numerous volcanic edifices associated with hydrothermal deposits^3–9^.

The Lau Basin–Tonga Arc system is characterized by rapid subduction and active back-arc extension, providing a favorable geodynamic environment for caldera formation, intrusive activity, and hydrothermal circulation^1,2^. Geodetic observations indicate that the Pacific plate subducts westward beneath the Indo-Australian plate at rates exceeding ~ 20 cm/yr in the northern Tonga region, among the fastest convergence rates globally^10^. Such rapid convergence has been linked to trench retreat and slab rollback, which promote vigorous back-arc extension and segmented spreading in the Lau Basin^2,11^. Back-arc spreading is accommodated by discrete spreading centers and rift zones, resulting in complex interactions among magmatism, faulting, and hydrothermal circulation^2^. Volcanic edifices in this arc–back-arc system commonly exhibit caldera collapse structures, ring faults, intrusive complexes, and hydrothermal alteration zones, reflecting the coupled effects of tectonic extension and arc-related magma supply^9,12,13^. These regional tectono-magmatic processes provide the structural and geodynamic framework for the development and evolution of seamounts such as TA12.

Hydrothermal circulation develops where seawater penetrates fractures along ridges and subduction-related structures, interacts with magma or mineral-rich substrates, and is subsequently discharged as heated fluids. This process leads to the precipitation of metallic minerals such as iron, manganese, and copper, forming seafloor hydrothermal deposits^14^. Previous geophysical investigations of submarine massive sulfide (SMS) deposits have employed bathymetric mapping, sonar backscatter analysis, geomagnetic anomaly surveys, sediment geochemistry, and seafloor imaging^15–21^. These studies have advanced knowledge of SMS morphology, mineralogy, and evolutionary history; however, they have generally not provided comprehensive three-dimensional (3D) representations of the internal structure of individual seamounts.

Magnetization vector inversion (MVI) provides a methodological advance by jointly considering induced and remanent magnetization^22–25^. Although while MVI has been widely applied in continental mineral exploration, its application to submarine volcanic systems remains comparatively limited. Recent studies integrating magnetic inversion with seismic profiling have demonstrated the potential of MVI to delineate magma migration pathways, intrusive features, and alteration zones^26^. Nevertheless, detailed three-dimensional imaging of seamount formation processes in arc-related submarine environments remains rare.

In contrast to previous magnetic investigations of TA12, which primarily relied on total-field anomaly mapping and qualitative structural interpretation, the present study applies full three-dimensional MVI to recover both the magnitude and direction of magnetization within a volumetric framework. By solving for the full magnetization vector, this approach reduces the ambiguity associated with purely induced-magnetization assumptions and enables a more rigorous assessment of structural controls on magnetization distribution. In addition, systematic sensitivity testing—including perturbations of geomagnetic field parameters and threshold criteria—was performed to assess the robustness of the first-order spatial magnetization patterns.

Building on this methodological framework, we focus on the the TA12 seamount in the Lau Basin. TA12 exhibits a prominent caldera with cone-shaped edifices and slope failures, providing an excellent natural laboratory for evaluating the utility of MVI in resolving submarine volcanic structure. We analyze multibeam bathymetry and shipborne magnetic data collected during the 2009 KIOST survey, applying IGRF correction, reduction to the pole (RTP), analytic signal transformation, and 3D MVI modeling^27^. By comparing the inversion results with published seismic profiles, we aims to: (i) reconstruct the 3D internal formation structure of TA12; (ii) assess alternative interpretations of the magnetic lows (e.g., hydrothermal alteration, gravitational collapse, and volcaniclastic infill); and (iii) propose a conservative three-stage evolutionary model consistent with the integrated geophysical evidence. More broadly, this study demonstrates how integrating three-dimensional MVI with complementary geophysical observations can improve the interpretation of submarine arc volcano structure where direct subsurface information is limited.

Through this approach, we emphasize both the opportunities and the limitations of applying MVI to submarine volcanic systems. The results illustrate how integrating full three-dimensional MVI with independent bathymetric and seismic constraints may provide a reproducible framework for seamount imaging, while also highlighting targets for future near-bottom surveys and resource assessments in back-arc basin environments.

## Methods

### Data acquisition

Bathymetric and magnetic surveys of the TA12 seamount were conducted aboard the R/V Onnuri, operated by the Korea Institute of Ocean Science and Technology (KIOST), on 8–10 October 2009. Bathymetric data were acquired using a 12 kHz-class Kongsberg EM120 multibeam echo sounder, whereas magnetic measurements were obtained using a high-sensitivity Overhauser-type marine magnetometer (0.01 nT sensitivity, 1 Hz sampling rate) towed approximately three vessel lengths astern to minimize ship-related magnetic noise. The survey grid comprised six latitudinal and six longitudinal profiles spaced at ~ 1 nautical mile (~ 1.85 km), covering both the summit and flanks of TA12 (Fig. 1).

Line levelling was performed using a crossover-based least-squares network adjustment implemented in the Oasis montaj Geophysics Levelling module (Seequent, a Bentley Systems company). This approach minimizes discrepancies at survey line intersections by estimating and removing systematic line-to-line offsets. Background regional trends were removed prior to inversion processing. Navigation was recorded using differential GPS with sub-meter accuracy, and all positions were processed in the WGS84 datum and UTM Zone 1S coordinate system.

Given the ~ 1.85 km line spacing, the effective lateral sampling interval limits resolvable wavelengths to approximately twice the line spacing (~ 3–4 km), consistent with Nyquist sampling considerations. Consequently, interpretations focus on kilometer-scale magnetization patterns rather than sub-kilometer features. Vertical resolution in potential-field inversion is inherently lower than horizontal resolution due to the decay of magnetic sensitivity with depth; therefore, the recovered depth extent of magnetized bodies should be regarded as a detectability limit rather than a sharp geological boundary.

During the survey period (8–10 October 2009), global Kp index values were generally ≤ 2, with short-lived peaks up to 4.5. No geomagnetic storm conditions (Kp ≥ 5) were recorded, indicating that the magnetic data were acquired under predominantly quiet to moderately disturbed geomagnetic conditions. Kp values were obtained from the GFZ Potsdam geomagnetic indices database^28^.

The total magnetic field data were corrected for the International Geomagnetic Reference Field (IGRF-13)^29^, evaluated for the survey epoch (2009), to remove the large-scale core field component.

Conventional diurnal variation correction based on a fixed land observatory was not applied because the survey area is located in a remote open-ocean setting, approximately 1200 km from the nearest permanent geomagnetic observatory. This large separation distance raises concerns regarding the spatial representativeness of short-period external field variations. Differences in temporal sampling resolution between observatory data and shipborne measurements may also introduce additional uncertainties.

Instead, following common practice in marine magnetic surveys conducted in remote oceanic regions^30,31^, crossover-based leveling and network adjustment were used to suppress residual long-wavelength temporal variations and systematic offsets between survey lines. Given the predominantly quiet geomagnetic conditions during data acquisition, these leveling procedures are considered sufficient to minimize residual temporal magnetic field effects in the present dataset.

### Total magnetic intensity and analytic signal

The raw magnetic data were corrected for the International Geomagnetic Reference Field (IGRF-13), evaluated for the survey epoch (2009), to remove the large-scale core field component. After removal of the IGRF field, the total-field magnetic anomaly ($\Delta T$) was calculated as the residual magnetic field associated with crustal sources. For further interpretation, the $\Delta T$ data were processed using reduction to the pole (RTP)^32^ and analytic signal transformations.

The RTP procedure re-centered the anomaly distribution by applying the regional geomagnetic inclination (− 42.8°) and declination (13.4°) corresponding to the survey period and location. At this latitude, RTP processing is not expected to introduce significant instability; rather, it improves the geometric centering of magnetic anomalies relative to their causative sources. RTP was applied solely as a qualitative visualization step to facilitate comparison with bathymetric structures and was not used to constrain or initialize the subsequent MVI inversion.

The analytic signal transformation highlights anomaly amplitude independent of magnetization direction and is therefore well suited to delineating the lateral extent of magnetic sources. Following MacLeod et al.^33^ and Roest et al.^34^, the analytic signal amplitude is defined as the root-sum-square of the three orthogonal spatial derivatives of the $\Delta T$ anomaly:

1 $${\mathrm{A}} = \sqrt {\left( {\frac{{\partial {\Delta }T}}{\partial x}} \right)^{2} + \left( {\frac{{\partial {\Delta }T}}{\partial y}} \right)^{2} + \left( {\frac{{\partial {\Delta }T}}{\partial z}} \right)^{2} }$$

where $\Delta T$ is the total-field magnetic anomaly. This transformation enhances edge detection of magnetic bodies and provides complementary information for structural interpretation alongside the subsequent inversion analysis. To support robust modeling, data error estimates were derived from repeat-line statistics and incorporated into inversion weighting functions.

### Magnetization vector inversion (MVI)

Magnetization Vector Inversion (MVI) is a three-dimensional inversion technique that simultaneously estimates both induced and remanent magnetization, in contrast to conventional magnetic inversions that typically assume a constant induced direction parallel to the Earth’s main field^25,35,36^. This capability is particularly important in submarine volcanic settings, where compositional variability and complex volcanic histories may produce spatial variations in magnetization direction. Unlike scalar susceptibility inversion, which assumes that magnetization is strictly parallel to the present geomagnetic field, MVI solves directly for the full magnetization vector (three independent components per voxel), allowing the magnetization direction to deviate from the ambient field and explicitly accounting for remanent contributions^25,37^.

From an inverse-theory perspective, MVI increases the number of model parameters from one susceptibility value per voxel to three independent magnetization components. The problem is therefore more underdetermined and requires regularization to obtain a stable and geologically meaningful solution. In this study, the inversion framework follows standard voxel-based potential-field inversion theory based on Tikhonov regularization^38^, in which the solution is obtained by minimizing a composite objective function that balances data misfit and model smoothness.

The forward problem can be expressed as:

2 $$d = Gm$$

where $d$ represents the observed total-field magnetic anomaly ($\Delta T$), G is the sensitivity (kernel) matrix relating subsurface sources to the data, and m is the concatenated magnetization vector model, with each voxel containing three independent Cartesian components (${M}_{x}$, ${M}_{y}$, ${M}_{z}$). The total magnetization can be decomposed as:

3 $$M = M_{ind} + M_{rem}$$

with ${M}_{ind}$ is aligned with the present geomagnetic field, and ${M}_{rem}$ represents remanent magnetization with independent amplitude and direction^25,35,36^.

The inversion minimizes an objective function defined as:

4 $$\phi \left( m \right) = { }\phi_{d} + { }\beta \phi_{m}$$

The normalized data misfit term is:

5 $$\phi_{d} = { }\frac{1}{N}\mathop \sum \limits_{i = 1}^{N} \left( {\frac{{d_{i}^{obs} - { }d_{i}^{pred} }}{{\sigma_{i} }}} \right)^{2}$$

and the model regularization term is:

6 $$\phi_{m} = \left\| {W_{m} m} \right\|^{2}$$

where, ${\sigma }_{i}$ denotes the assigned data uncertainty, ${W}_{m}$ is the model weighting operator imposing spatial smoothness, and $\beta$ is the trade-off parameter controlling the balance between data fit and model complexity^25,35^.

A uniform absolute data uncertainty of 5 nT was assigned to all magnetic observations based on repeat-line (cross-over) statistics. Cross-over differences were calculated at line intersections and statistically summarized to estimate survey noise. The standard deviation of these differences was approximately ± 5 nT, and this value was adopted as ${\sigma }_{i}$ for all observations, consistent with standard potential-field inversion practice^25,39^.

The inversion was configured to achieve a normalized misfit (${\phi }_{d}\approx 1$), corresponding to a statistically consistent fit within the prescribed noise level. The regularization parameter $\beta$ was determined using the VOXI automated trade-off adjustment procedure, which iteratively updates $\beta$ until the target misfit level is reached. This approach is consistent with standard Tikhonov regularization strategies for potential-field inversion.

In contrast to conventional scalar or FFT-based inversions, which assume a fixed magnetization direction, MVI allows independent recovery of magnetization magnitude and direction, providing a flexible framework for modeling volcanic systems where remanent magnetization may be significant^25,35,36^.

MVI was implemented using the VOXI Earth Modelling system (Seequent, a Bentley Systems company), a voxel-based three-dimensional potential-field inversion platform that solves for model parameters within a discretized volumetric grid using a Tikhonov-regularized objective function^40^. In the MVI configuration, three magnetization components were recovered independently in each voxel. The inversion incorporated data-error weighting, depth weighting, and smoothness constraints within an iterative optimization scheme.

The active inversion domain comprised 41 × 43 × 27 cells in the x, y, and z directions, with horizontal cell dimensions of 250 m × 125 m. The model extended from the sea surface to approximately 6315 m depth, encompassing the full volume of TA12. A depth-weighting function was applied to compensate for the decay of magnetic sensitivity with depth, reducing bias toward shallow sources while improving recovery of deeper magnetized structuresp^41^. Nevertheless, vertical resolution remains inherently lower than horizontal resolution.

Regularization was imposed using an L2-norm smoothness constraint applied equally in horizontal and vertical directions. No reference model or hard bounds were imposed, and the starting model consisted of zero magnetization in all cells. No directional constraints or preferred magnetization directions were applied; the inversion was performed in a fully free MVI mode. To reduce boundary effects, the active domain was embedded within a larger padded computational grid, although only the central active region was interpreted.

The inversion converged after 11 iterations (Supplementary Table S1), achieving a final normalized data misfit of 0.998. A summary of the principal inversion parameters, model configuration, and regularization settings is provided in Supplementary Table S5. Although magnetic inversion is inherently non-unique^42^, the recovered model represents a geologically plausible magnetization distribution that is consistent with the observed magnetic data and independently interpreted bathymetric and seismic structures.

The resulting three-dimensional model provides both magnetization magnitude and directional components (declination and inclination) across TA12. To facilitate reproducibility, all processed magnetic grids, inversion parameters, and visualization scripts used in this study have been archived in a public Zenodo repository and are available through the Data and Code availability statements.

## Results

### Bathymetric properties

**Fig. 2:**
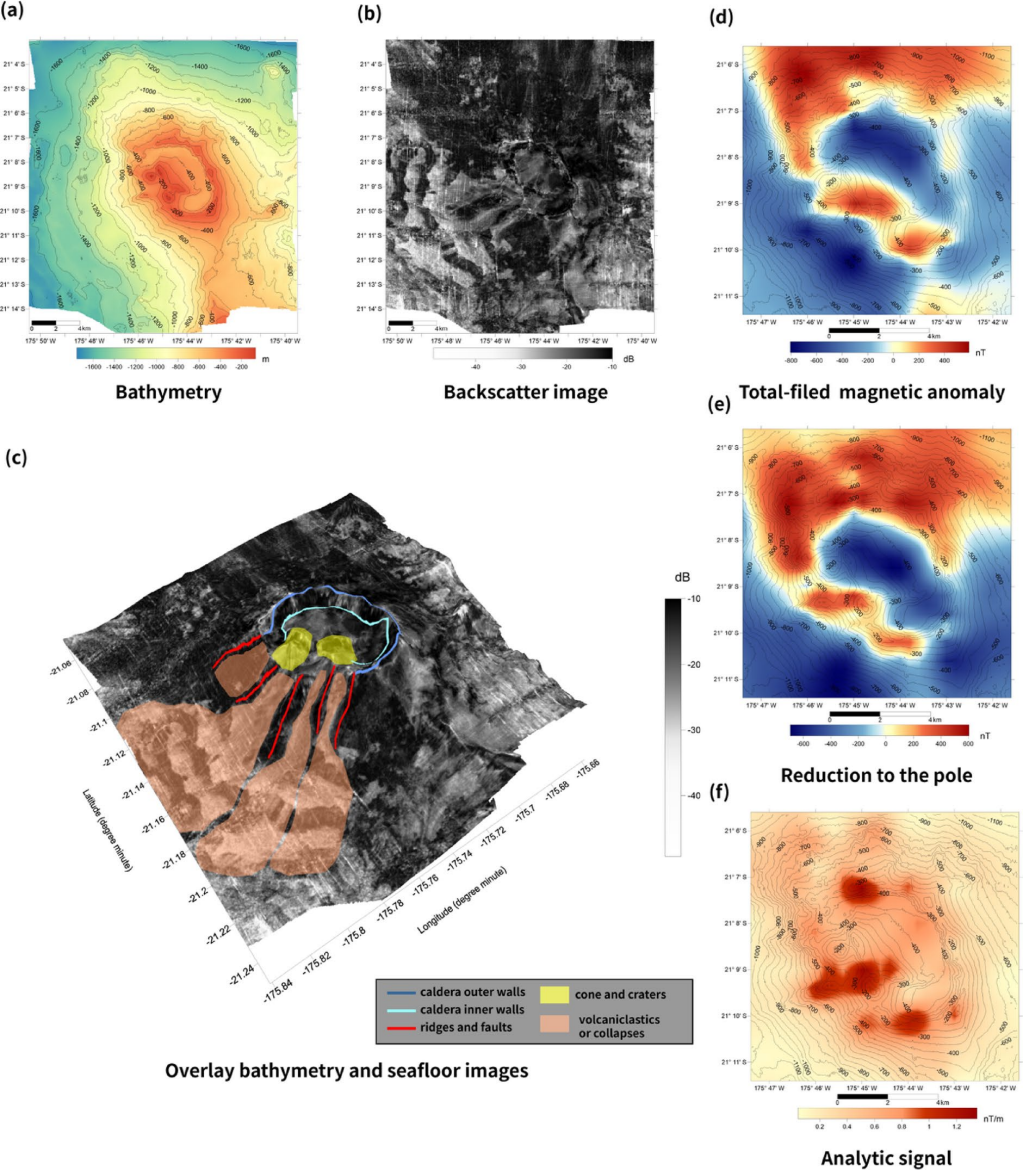
Bathymetric and magnetic datasets of the TA12 seamount. (**a**) Two-dimensional bathymetric contour map. (**b**) Acoustic backscatter image. (**c**) Backscatter image with geological and tectonic features overlaid on the 3D bathymetric surface. (**d**) Total-field magnetic anomaly ($\Delta T$**,** nT) after IGRF correction. (**e**) Reduction-to-the-pole (RTP) anomaly calculated using inclination -42.8° and declination 13.4°. (**f**) Analytic signal amplitude (nT/m). Figure was generated using Surfer 13 (Golden Software, https://www.goldensoftware.com/) for data visualization, and Adobe Illustrator 2026 (Adobe, https://www.adobe.com/) for final figure composition.

The TA12 seamount is a volcanic edifice characterized by a single main summit and an elliptical caldera elongated in a northwest–southeast direction (Fig. 2a). Two small cone-shaped features occur within the caldera depression. The caldera floor deepens to a water depth of ~ 400 m, compared to ~ 200 m along the rim, resulting in a total relief of ~ 200 m between the depression and rim. The base of the seamount lies ~ 1600 m below sea level, and the shallowest summit reaches a water depth of ~ 200 m. Both cones display small summit craters, suggesting constructional activity following the main caldera-forming episode.

The southwestern slope hosts a submarine canyon and exhibits lower backscatter intensity relative to other flanks (Fig. 2b). Integration of bathymetric and backscatter data reveals features interpreted as large-scale mass-transport deposits along the southwestern base of the edifice (Fig. 2c). These deposits may reflect collapse of the western caldera wall, potentially involving lava and volcaniclastic material; however, tectonic slope failure cannot be excluded. Two small cones with craters adjacent to the western caldera wall indicate localized post-collapse eruptive activity within the depression.

The caldera rim is defined by steep inner and outer walls with cliff-like morphology. Such geometry is consistent with fault-bounded collapse following magma withdrawal and subsequent eruptive phases, as described for comparable submarine volcanic systems^12,43^. The structural configuration implies the presence of ring-fault–like features along the caldera margin. All morphological interpretations presented here should be considered within the resolution limits imposed by the ~ 1 nautical mile survey line spacing.

### Magnetic properties

The total-field magnetic anomaly (ΔT) over TA12 ranges from approximately − 805 to + 592 nT (Fig. 2d). Although lower than the maximum amplitudes reported for intra-arc seamounts such as Brothers volcano (up to ~  ± 1500 nT)^12^, these values suggest the presence of significant magnetization contrasts within TA12. Elevated anomalies are concentrated along the caldera rim and adjacent slopes, whereas lower values occur within the depression.

After RTP, anomaly patterns become more symmetrically aligned with bathymetric structures, with positive amplitudes concentrated along the northern and western rim sectors and more subdued values within the caldera depression (Fig. 2e).

The analytic signal amplitude delineates source boundaries more clearly, emphasizing the steep inner walls of the caldera and the northern, western, and southern rim sectors (Fig. 2f). Owing to its relative insensitivity to magnetization direction, the analytic signal is widely used for edge detection of magnetic sources^34^. At TA12, enhanced amplitudes along the caldera rim are consistent with magnetized materials concentrated along structurally controlled margins. The relatively low amplitudes within the depression may reflect several processes, including: (i) hydrothermal alteration of titanomagnetite to weakly magnetic phases^16,17^; (ii) redistribution of volcaniclastic material following caldera collapse; or (iii) structural disruption of magnetized units^12,13,43,44^.

Low-magnetization zones comparable to those observed at TA12 have been documented in hydrothermal systems along mid-ocean ridges and volcanic arcs^16,17^. However, the available magnetic data alone do not uniquely constrain the relative contributions of alteration, mass wasting, or primary lithologic variability. Alternative scenarios, such as tectonic slope failure, non-uniform intrusion pathways, or development of monogenetic vents, therefore remain possible^45–50^.

Resolution limitations must also be considered. Given the ~ 1.85 km line spacing, resolvable wavelengths are expected to exceed a few kilometers based on sampling considerations. Accordingly, sub-kilometer-scale features are unlikely to be robustly imaged in the present dataset^50^. The observed anomaly patterns primarily reflect kilometer-scale volcanic structure rather than deposit-scale features. Cross-comparison of bathymetry and published seismic profiles indicates spatial correspondence between rim-focused anomalies and structurally defined sectors of the edifice.

Stability tests of the RTP correction were conducted to assess the sensitivity of the anomaly patterns to uncertainties in geomagnetic field parameters. RTP maps were recalculated using inclination and declination values perturbed by ± 10° relative to the reference field (I =  − 42.8°, D = 13.4°). Across all tested cases, the first-order distribution of magnetic anomalies—including high amplitudes along the caldera rim and subdued anomalies within the depression—remains largely unchanged (Supplementary Fig. S1). Quantitative comparison confirms that the spatial overlap of rim-focused anomaly maxima is preserved within a narrow range across perturbation scenarios (Supplementary Table S2). These results indicate that the first-order spatial distribution of magnetic anomalies is not highly sensitive to moderate variations in geomagnetic field direction.

To evaluate potential topographic effects, the observed $\Delta T$ field was compared with bathymetric slope derived from the same grid. The resulting correlation is weak and statistically insignificant (Supplementary Fig. S2), suggesting that the rim-focused anomalies are not solely controlled by bathymetric gradients but reflect subsurface magnetization contrasts.

### MVI modeling

**Fig. 3:**
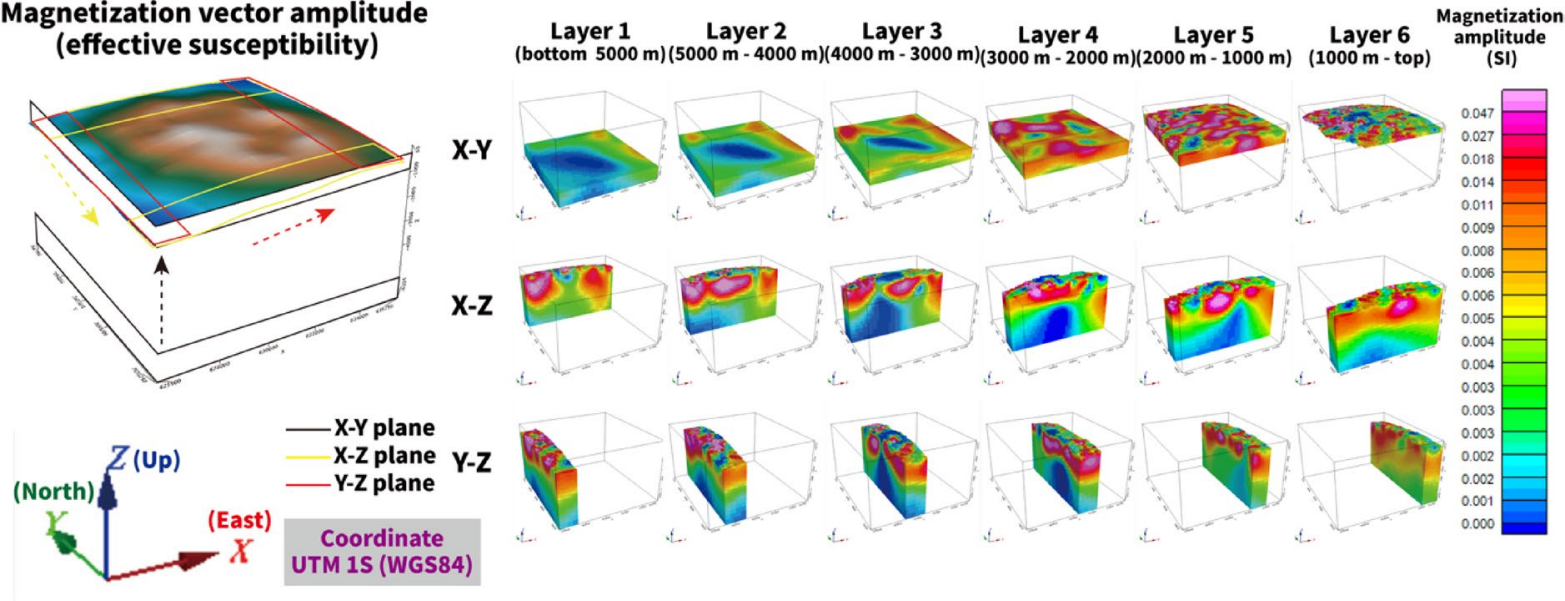
Magnetization vector amplitude derived from three-dimensional magnetization vector inversion (MVI) of the TA12 seamount. Horizontal slices at selected depths and vertical cross-sections (X–Z and Y–Z planes) show the distribution of magnetization amplitude expressed as effective susceptibility (SI). Elevated amplitudes are concentrated along the caldera rim and within the shallow subsurface, whereas lower amplitudes occur at depths greater than approximately 3 km bsl. Axes are shown in UTM Zone 1S (WGS84). Figure was generated using Oasis montaj 2025.1 (Seequent, https://www.seequent.com/) for data processing and visualization, and Adobe Illustrator 2026 (Adobe, https://www.adobe.com/) for final figure composition.

The three-dimensional MVI results resolve systematic variations in magnetization within TA12 (Fig. 3). Magnetization amplitude is generally higher at depths shallower than approximately 3 km below sea level (bsl), whereas lower amplitudes dominate at greater depths (> 3 km bsl). On horizontal slices, enhanced magnetization is concentrated along the caldera rim and on the northern and southern flanks. Within the caldera depression, zones of reduced magnetization spatially coincide with mass-wasting features and volcaniclastic deposits inferred from bathymetric observations.

At shallower depths (~ 1–2 km bsl), localized magnetization highs occur beneath the caldera depression and along the inner rim. These features may be consistent with intrusive bodies and subsequent volcanic activity. Smaller-scale magnetization highs within the depression, particularly near the two cones, may reflect localized lava or pyroclastic emplacement.

**Fig. 4:**
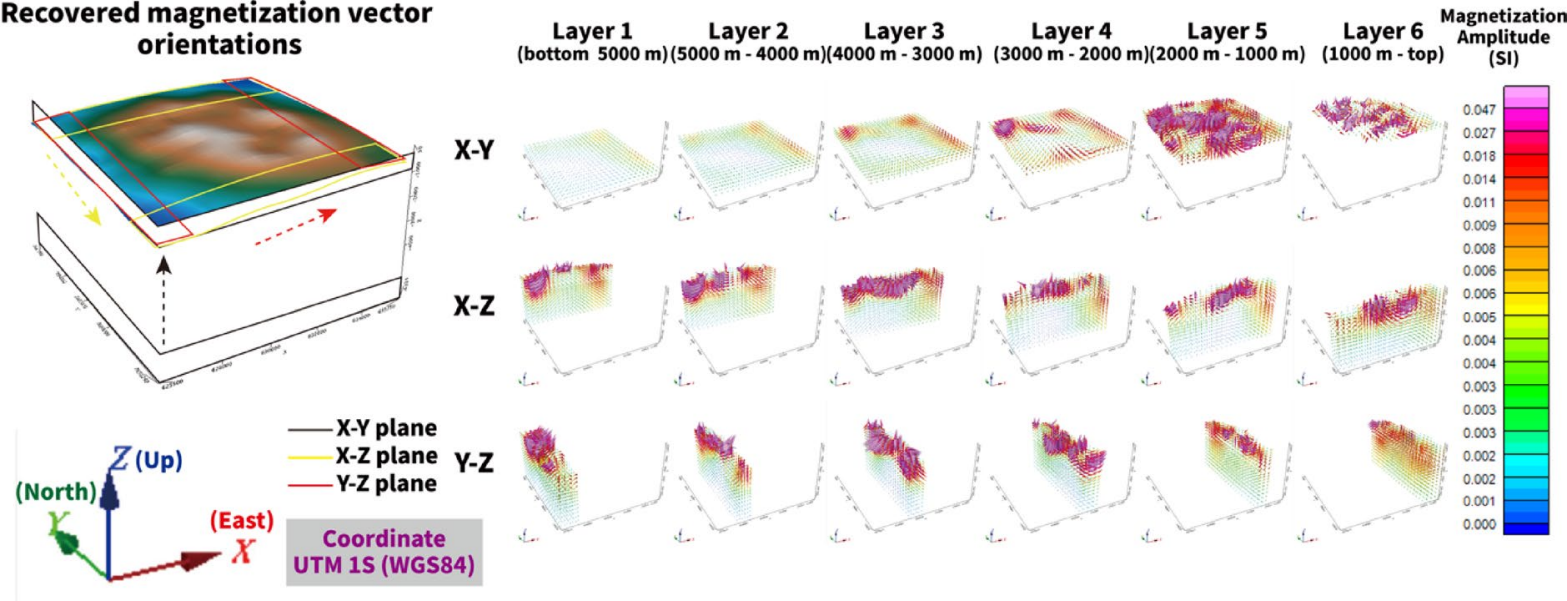
Recovered magnetization vector orientations from three-dimensional magnetization vector inversion (MVI) of the TA12 seamount. The vectors are derived from the same MVI solution shown in Figure. Horizontal and vertical slices correspond to those in Figure. Colors represent magnetization amplitude (effective susceptibility, SI), and arrows indicate magnetization vector direction, with arrow length scaled proportionally to amplitude. Axes are shown in UTM Zone 1S (WGS84). Figure was generated using Oasis montaj 2025.1 (Seequent, https://www.seequent.com/) for data processing and visualization, and Adobe Illustrator 2026 (Adobe, https://www.adobe.com/) for final figure composition.

In addition to magnetization amplitude, the MVI model recovers spatial variations in magnetization direction (declination and inclination), as shown in Fig. 4. The recovered magnetization vectors are not uniformly aligned with the present geomagnetic field, indicating spatial variability in magnetization direction. This variability supports the use of full vector inversion and suggests that remanent components contribute to the overall magnetization structure.

Vertical cross-sections further illustrate these patterns. On the X–Z and Y–Z planes, elevated magnetization is observed beneath the northeastern flank and central caldera region, forming arcuate features that extend toward the seafloor. In contrast, reduced amplitudes dominate deeper levels. These deeper low-amplitude zones may reflect either less magnetized basement or reduced sensitivity of the magnetic data with increasing depth.

The distribution of voxel susceptibility values derived from the MVI model is strongly skewed toward low amplitudes (Supplementary Fig. S3), providing a quantitative basis for selecting susceptibility thresholds in subsequent sensitivity analyses. The reported susceptibility values represent effective susceptibilities calculated by projecting the recovered magnetization vectors onto the present ambient geomagnetic field direction. The inversion itself directly recovers the full magnetization vector (${M}_{x}$, ${M}_{y}$, ${M}_{z}$) in each voxel.

**Fig. 5:**
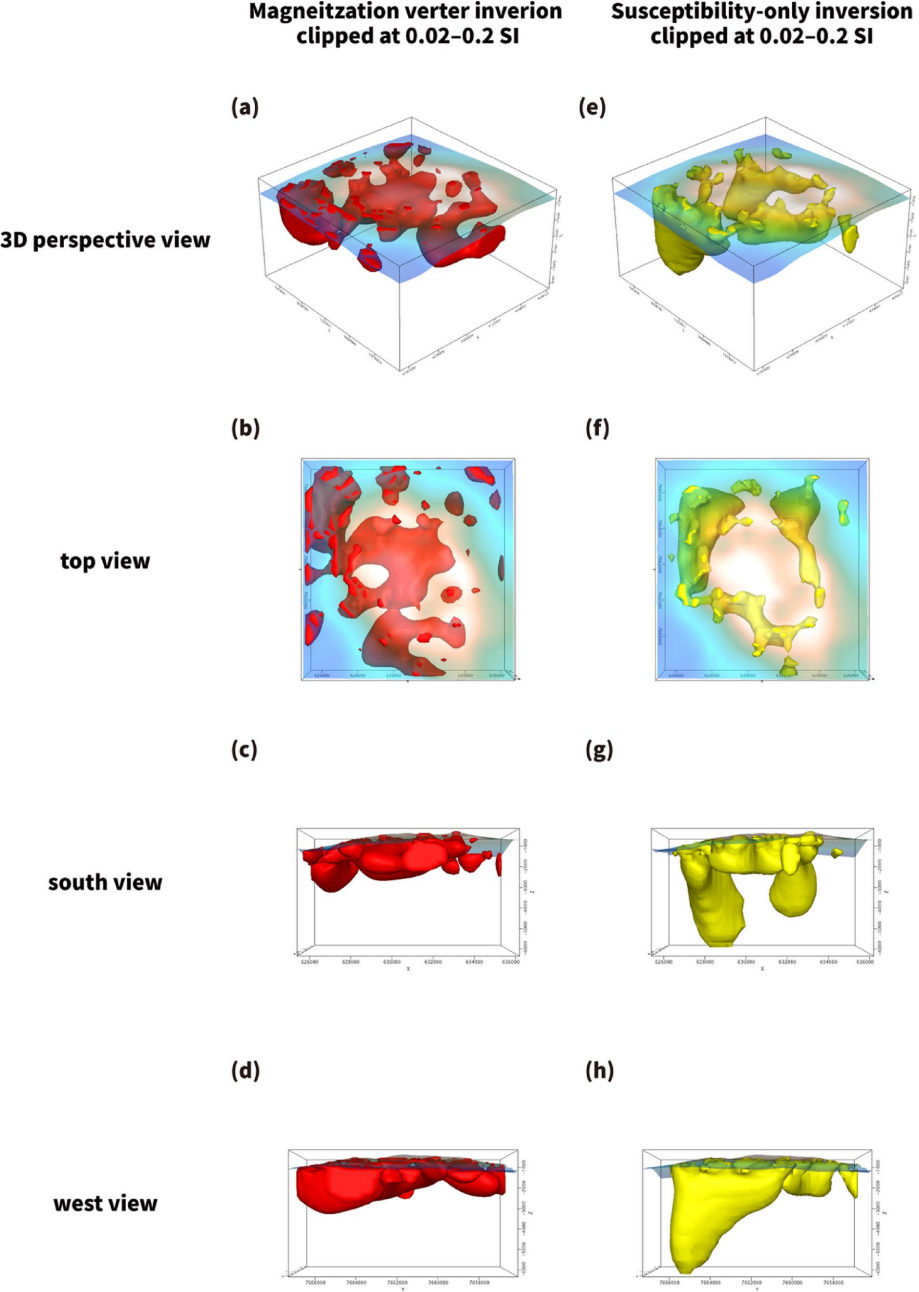
Comparison between MVI and susceptibility-only inversion results for TA12. (**a**–**d**) Three-dimensional isosurface views (0.02–0.2 SI) of the full MVI model, which recovers both induced and remanent magnetization components, shown in perspective, top, south, and west views. (**e**–**h**) Corresponding views of the susceptibility-only model assuming purely induced magnetization aligned with the present geomagnetic field. Compared with the susceptibility-only result, the MVI model resolves sharper and more spatially focused rim-associated magnetization patterns, reflecting differences in the recovered magnetization direction. Figure was generated using Oasis montaj 2025.1 (Seequent, https://www.seequent.com/) for data processing and visualization, and Adobe Illustrator 2026 (Adobe, https://www.adobe.com/) for final figure composition.

For comparison, a susceptibility-only inversion, assuming purely induced magnetization, was also performed (Fig. 5). Although this approach reproduces the first-order anomaly distribution, it yields smoother and more symmetric patterns without directional information. In contrast, the full MVI solution allows independent recovery of magnetization direction and produces more spatially focused rim-associated anomalies. The differences between the two models suggest that remanent magnetization contributes to the observed anomaly field and influences the spatial expression of magnetization contrasts.

The quality of the inversion fit is demonstrated by comparison between the observed $\Delta T$, the forward-calculated response of the recovered model, and their residual (Supplementary Fig. S4). Residuals are generally low in amplitude and lack coherent spatial structure, indicating that the MVI model reproduces the first-order magnetic anomaly field within the prescribed noise level.

**Fig. 6:**
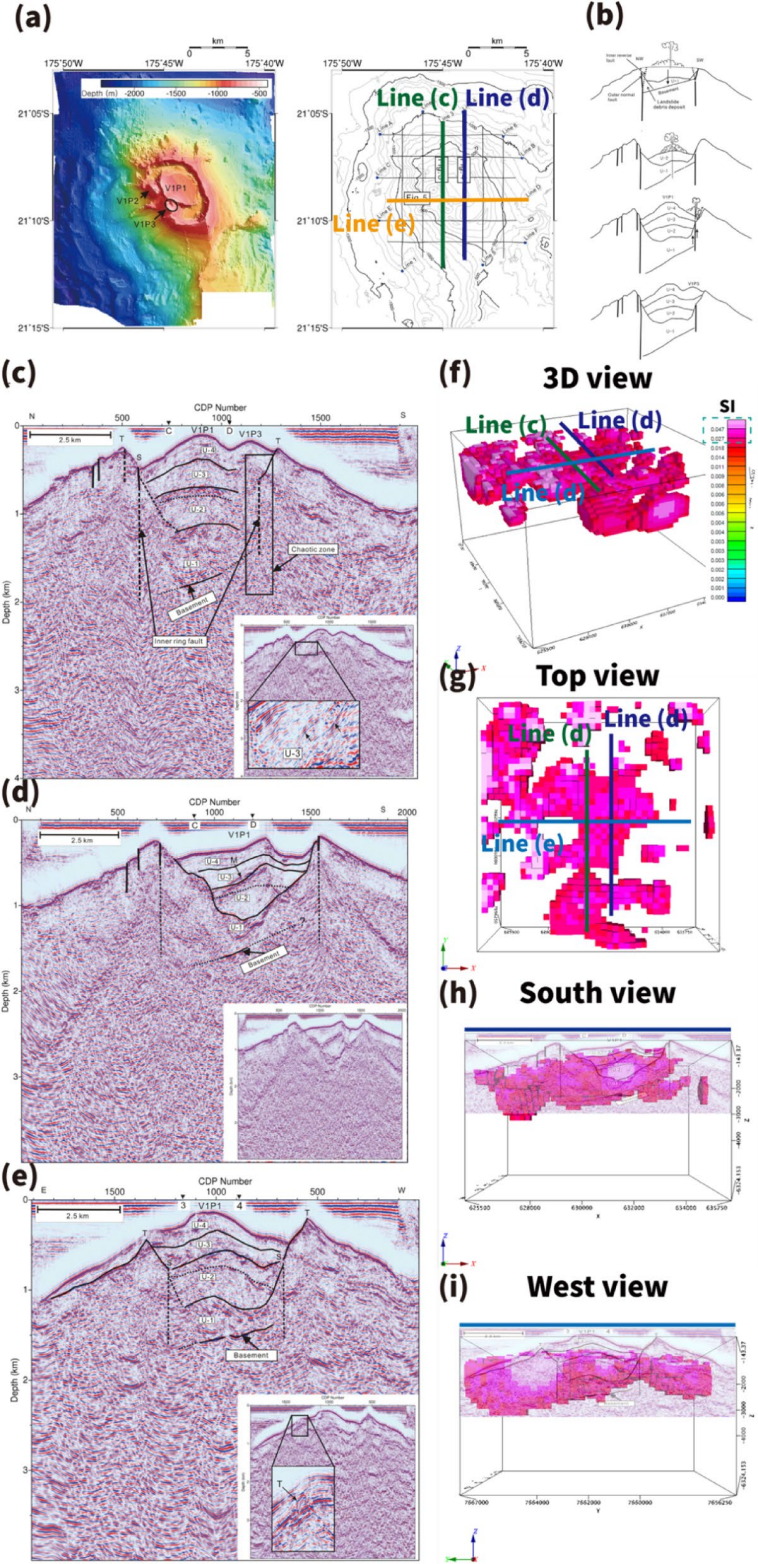
Comparison of clipped MVI susceptibility distribution (0.02–0.2 SI) with published seismic profiles of TA12. (**a**) Bathymetric map of TA12 with seismic track lines, modified from ref. 52. (**b**) Conceptual formation model inferred from seismic interpretation, modified from ref. 52. (**c**–**e**) Seismic reflection profiles showing caldera-related structures, including ring faults, chaotic facies, and a shallow basement interface near ~ 2 km bsl, modified from ref. 52. (**f**–**i**) MVI-derived magnetic susceptibility distributions clipped to 0.02–0.2 SI, shown as a 3D perspective view and corresponding X–Y, Y–Z, and X–Z slices. The spatial correspondence supports fault-controlled magma emplacement and post-caldera intrusive activity. Because the seismic profiles were acquired independently from the magnetic survey, the comparison is intended to be qualitative and focuses on first-order structural correspondence rather than exact positional matching. Figure was generated using Oasis montaj 2025.1 (Seequent, https://www.seequent.com/) for data processing and visualization, and Adobe Illustrator 2026 (Adobe, https://www.adobe.com/) for final figure composition. Panels (**a**–**e**) are reproduced from previously published studies, and panels (**f**–**i**) include a combination of newly generated and reproduced elements.

Seismic data were not incorporated as constraints in the inversion and were used only for qualitative comparison. Comparison with published seismic profiles^51^ indicates spatial correspondence between magnetization highs and shallow structural discontinuities, including reflectors interpreted as basement interfaces and caldera-related ring faults at approximately 2 km bsl. Regions of elevated susceptibility (0.02–0.2 SI) are aligned with structurally defined sectors of the caldera rim (Fig. 6).

To evaluate the robustness of these spatial patterns, susceptibility thresholds of 0.015, 0.020, and 0.030 SI were tested. The rim-focused distribution remains stable across threshold variations (Supplementary Fig. S5; Supplementary Tables S3–S4), indicating that the primary structural pattern is not highly sensitive to cutoff selection.

Because seismic navigation data were unavailable, the comparison is based on published sections and associated geographic information. The estimated horizontal positional uncertainty is on the order of several hundred meters to ~ 1 km, and interpretations are therefore made at kilometer scale.

Although coherent magnetization patterns are resolved to depths of approximately 3 km bsl, the apparent lower boundary of high-amplitude zones should not be interpreted as a sharp geological interface. Magnetic sensitivity decreases with depth despite depth weighting, and the recovered depth extent of magnetized bodies reflects a detectability limit rather than a definitive petrological boundary.

Resolution constraints must also be considered. With ~ 1.85 km line spacing, resolvable wavelengths are expected to exceed ~ 2–3 km based on Nyquist considerations. Accordingly, the MVI model primarily resolves kilometer-scale volcanic structures rather than sub-kilometer intrusive conduits or individual hydrothermal pathways.

Some magnetization highs, particularly on the flanks of the edifice, do not correspond to distinct features in the available seismic profiles. This mismatch likely reflects differences in physical sensitivity and resolution between magnetic and seismic methods, as magnetic data integrate volumetric magnetization contrasts over broader depth ranges. Higher-resolution near-bottom surveys would be required to further evaluate these localized features.

## Discussion

The combined analysis of magnetic anomalies, RTP-stabilized fields, analytic signal amplitudes, and MVI results reveals a robust first-order relationship between magnetization patterns and the structural framework of the TA12 seamount. Rim-focused magnetic highs are consistently observed across independent magnetic representations and remain stable under moderate perturbations of geomagnetic field parameters and susceptibility thresholds. These convergent observations indicate that the recovered magnetization patterns primarily reflect subsurface structure rather than processing artifacts. In this study, “first-order” patterns refer to magnetization features that (i) remain stable across RTP perturbation tests and susceptibility threshold variations, and (ii) extend laterally over kilometer-scale dimensions consistent with the resolution limits imposed by the survey geometry.

In addition to resolving magnetization amplitude, the full MVI solution provides directional information that is not available in induced-only susceptibility inversion. When magnetization is constrained to align with the present geomagnetic field, the resulting models tend to produce smoother and more symmetric patterns. By contrast, the vector-based inversion allows voxel-wise deviations in magnetization direction and yields more spatially focused rim-associated anomalies. These differences suggest that non-induced (potentially remanent) components contribute to the observed anomaly field and influence the recovered geometry of magnetization contrasts. The comparison therefore highlights the added interpretive value of full vector inversion in structurally complex submarine volcanic settings.

At the spatial resolution of the present shipborne survey, the MVI model is best interpreted as imaging the large-scale volcanic architecture of TA12 rather than discrete intrusive bodies or individual hydrothermal conduits. The concentration of magnetization along the caldera rim and shallow subsurface is consistent with structurally controlled magma emplacement and post-caldera intrusive reactivation^47^, as inferred from bathymetric and previously published seismic observations. The qualitative correspondence between rim-focused magnetization highs and seismic discontinuities interpreted as basement interfaces or ring-fault structures supports a structural control on magma localization, although the kilometer-scale positional uncertainty of the seismic sections requires conservative interpretation.

The final evolutionary stage of TA12 is characterized by reduced magnetization within the caldera depression and along fault-bounded sectors. The principal geophysical observation is therefore the spatially coherent decrease in magnetization and effective susceptibility in structurally defined regions. Magnetization loss in submarine volcanic systems is commonly associated with hydrothermal alteration, in which high-temperature fluids transform titanomagnetite into weakly magnetic phases, thereby reducing bulk magnetization^16,17^. At TA12, low-susceptibility zones spatially coincide with caldera-related ring faults, mass-wasting deposits, and chaotic seismic facies, suggesting that post-collapse faulting may have facilitated fluid circulation. Comparable spatial relationships among structural discontinuities, hydrothermal pathways, and magnetization reduction have been documented at Brothers volcano and other volcanoes along the Tonga–Kermadec arc^44^.

Mechanical disruption and redistribution of volcaniclastic material following caldera collapse may also contribute to the observed magnetization decrease^13^. Because magnetic inversion is inherently non-unique, and because the available data do not uniquely resolve mineralogical alteration, these mechanisms cannot be fully distinguished. Nevertheless, the spatial correspondence between magnetization lows and structurally interpreted fault zones suggests that hydrothermal alteration may represent a dominant, though not exclusive, control on the reduced magnetization within the caldera.

Resolution constraints must also be acknowledged. Given the survey geometry, the model primarily resolves kilometer-scale structures, and smaller-scale features may remain undetected. Accordingly, the recovered model should be regarded as a geologically plausible representation of magnetization contrasts that is consistent with the available magnetic, bathymetric, and independent seismic information, rather than as a unique structural solution.

Differences in spatial resolution and physical sensitivity between magnetic and seismic methods further influence cross-dataset comparisons. Some magnetization highs, particularly on the flanks of the seamount, do not correspond to distinct features in the available seismic profiles. This mismatch likely reflects the volumetric sensitivity of magnetic data compared with the wavelength-dependent response of seismic methods^50^.

In a broader regional context, the magnetization patterns observed at TA12 are consistent with tectono-magmatic processes operating throughout the Lau Basin–Tonga arc system, where back-arc extension and caldera collapse commonly promote intrusive reactivation and hydrothermal circulation^47^. Similar rim-focused magnetization highs and central magnetization lows have been documented at submarine arc volcanoes^12^ in the Kermadec, Mariana, and Izu–Bonin arcs. Comparable applications of marine magnetic analysis in the Tyrrhenian Sea and the Calabrian Arc have likewise demonstrated that intrusive domains and hydrothermal modification can generate systematic magnetization contrasts^52^. The spatial consistency of these magnetic–structural relationships suggests that the patterns observed at TA12 reflect processes characteristic of arc-related submarine volcanism^12^.

**Fig. 7:**
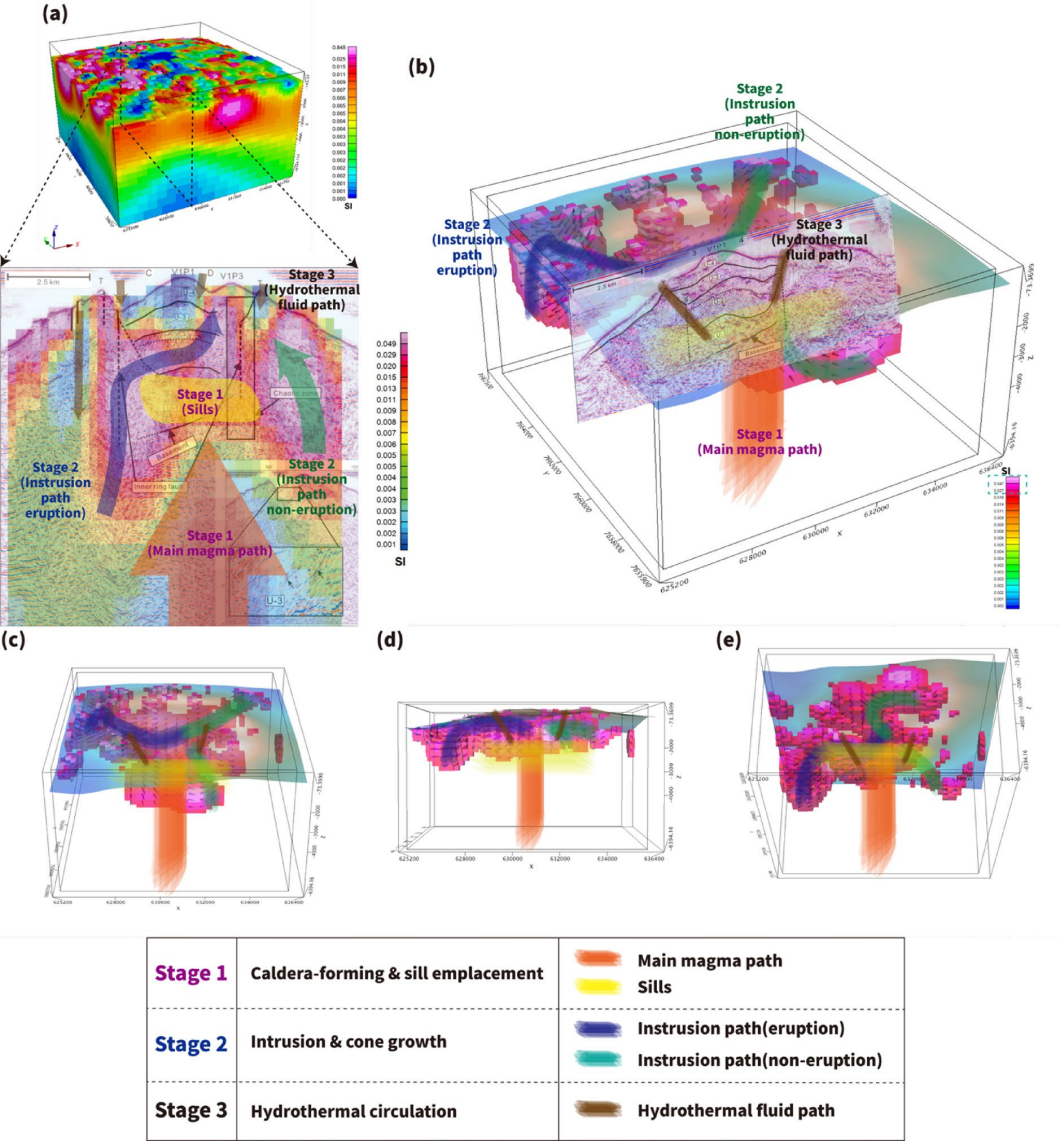
Conceptual three-stage evolutionary model of the TA12 seamount inferred from integrated MVI-derived magnetization patterns and seismic interpretations. (**a**) Conceptual illustration of the three-stage evolution (Stages 1–3) based on comparison between MVI-derived magnetization patterns and seismic cross-sections. Stage 1 represents a caldera-forming eruption with large-scale collapse and mass wasting, where the orange zone indicates the principal magma ascent pathway and the yellow body represents a shallow magma reservoir. Stage 2 involves renewed intrusive activity along caldera-related ring faults (interpreted as predominantly normal faults), with blue pathways indicating magma intrusions that reached the surface and green pathways representing non-eruptive shallow intrusions. Stage 3 corresponds to progressive structural modification and fault-controlled hydrothermal circulation, where brown zones indicate alteration and structural reorganization leading to localized reduction of magnetization. (**b**) Three-dimensional conceptual model illustrating the same three-stage evolution (Stages 1–3), showing the spatial distribution of magma pathways, intrusive bodies, and hydrothermal alteration zones. (**c**–**e**) Alternative viewing angles of the three-dimensional model shown in (**b**), provided to better illustrate the spatial geometry of magma pathways, structural features, and hydrothermal alteration zones. Black lines denote interpreted fault structures. The model is conceptual and not to scale. Figure was generated using Oasis montaj 2025.1 (Seequent, https://www.seequent.com/) for data processing and visualization, and Adobe Illustrator 2026 (Adobe, https://www.adobe.com/) for final figure composition. Some elements are adapted from previously published studies.

Taken together, the integrated magnetic, bathymetric, and published seismic observations motivate a conservative, conceptual three-stage evolutionary model for TA12 (Fig. 7). In this conceptual framework, (i) caldera collapse and associated mass-wasting processes are interpreted to have formed the primary depression; (ii) structurally controlled intrusions and renewed cone growth may have contributed to the shallow rim-associated magnetization highs; and (iii) fault-guided hydrothermal circulation and structural modification likely contributed to localized magnetization reduction within the caldera depression. This model is presented as an interpretative synthesis of the available geophysical evidence rather than as a uniquely constrained temporal reconstruction.

Despite the inherent limitations of marine magnetic resolution and inversion non-uniqueness, the combined use of full three-dimensional MVI, systematic sensitivity testing, and independent seismic comparison provides a potentially reproducible framework for assessing first-order internal structures of submarine arc volcanoes^25^. The structured workflow adopted here offers a transferable methodological template for investigating other poorly sampled volcanic systems and for guiding future near-bottom geophysical surveys aimed at resolving smaller-scale intrusive and hydrothermal features.

## Conclusions

- By integrating shipborne magnetic data with multibeam bathymetry, analytic signal analysis, RTP processing, and three-dimensional magnetization vector inversion (MVI), we constrained the first-order three-dimensional internal structure of the TA12 seamount in the Lau Basin.
- The observed rim-focused magnetization highs and reduced magnetization within the caldera depression were systematically evaluated. Among the considered mechanisms, hydrothermal alteration along caldera-related structures provides the most consistent explanation for the magnetic lows, supported by published seismic constraints, although alternative processes cannot be entirely excluded.
- At the resolution of the available data, the MVI results are best interpreted as imaging the large-scale volcanic structure of TA12 rather than discrete intrusive bodies.
- Integration of magnetic and seismic observations supports a conservative three-stage evolutionary model involving caldera collapse, subsequent intrusive reactivation, and later fault-controlled hydrothermal modification.
- These results provide a reproducible methodological framework for investigating poorly sampled submarine arc volcanoes by combining full three-dimensional MVI with complementary geophysical constraints, while acknowledging the inherent resolution limits and non-uniqueness of magnetic inversion. The ability to recover magnetization directions further improves interpretation compared with induced-only inversions.

## Supplementary Information

Below is the link to the electronic supplementary material.

Supplementary Material 1

Supplementary Material 2

## Data Availability

The processed shipborne magnetic anomaly grids, multibeam bathymetry datasets, RTP-transformed data, analytic signal maps, and final three-dimensional MVI model outputs generated during this study are publicly available in the Zenodo repository at (10.5281/zenodo.7483117).
