# Supplementary material for: Three dimensional magnetization structure of the Tofua Arc 12 seamount constrained by magnetization vector inversion
Source: Sci Rep. 2026 Apr 3;16:15960. doi: 10.1038/s41598-026-46834-x (PMC13194782; doi:10.1038/s41598-026-46834-x)
Supplement: Supplementary file 1 — Supplementary Material 1 [file 41598_2026_46834_MOESM1_ESM.docx]

**Supplementary Table S1 | Inversion convergence history and regularization scaling parameters for the MVI model.**

RegScal: regularization scaling factor; DataFit: normalized χ² misfit ($\phi_{d}$); ModelNorm: L2 model norm ($\phi_{m}$); Time: cumulative runtime in minutes.

| **nx** | **ny** | **nz** | **nx*ny*nz** | **ndata** | **nproc** | **Iter** | **RegScal** | **DataFit** | **ModelNorm** | **Time(min)** |
| --- | --- | --- | --- | --- | --- | --- | --- | --- | --- | --- |
| 51 | 53 | 32 | 86496 | 702 | 8 | 0 |  | 2464 | 0 | 0 |
|  |  |  |  |  |  | 1 | 2022000 | 46.0010 | 0.0335 | 1.12 |
|  |  |  |  |  |  | 2 | 252750 | 3.5213 | 0.0588 | 2.81 |
|  |  |  |  |  |  | 3 | 63188 | 0.7873 | 0.0771 | 6.08 |
|  |  |  |  |  |  | 4 | 78840 | 0.9902 | 0.0740 | 8.26 |
|  |  |  |  |  |  | Updating the IterReWeighting ... | | | |  |
|  |  |  |  |  |  | 5 | 78840 | 13.5680 | 0.1307 | 9.96 |
|  |  |  |  |  |  | 6 | 9855 | 0.9376 | 0.2069 | 13.22 |
|  |  |  |  |  |  | 7 | 10361 | 0.9929 | 0.2048 | 15.37 |
|  |  |  |  |  |  | 8 | 10361 | 18.2730 | 0.6621 | 17.06 |
|  |  |  |  |  |  | Updating the IterReWeighting ... | | | |  |
|  |  |  |  |  |  | 9 | 1295 | 1.2803 | 0.7408 | 21.35 |
|  |  |  |  |  |  | 10 | 916 | 0.8731 | 0.7578 | 25.63 |
|  |  |  |  |  |  | 11 | 1036 | 0.9980 | 0.7520 | 29.4 |

**Supplementary Table S2 | Quantitative comparison of RTP stability tests.**

Spatial overlap metrics for rim-focused magnetic anomaly patterns across RTP realizations with perturbed geomagnetic field parameters. Jaccard index and percentage overlap are computed relative to the reference RTP solution.

| **Case** | **Jaccard with Analytic Signal(%)** | **Overlap with Reference RTP(%)** |
| --- | --- | --- |
| RTP D −10° | 15.8974359 | 85.84070796 |
| RTP D +10° | 11.88118812 | 81.4159292 |
| RTP I −10° | 11.33004926 | 73.45132743 |
| RTP I +10° | 18.32460733 | 84.07079646 |

**Supplementary Table S3 | Statistical summary of voxel susceptibility values from the MVI model.**

| Parameter | Value |
| --- | --- |
| N (voxels) | 41,095 |
| Mean | 0.0114 |
| Std. Dev. | 0.0190 |
| Min | 0.000026 |
| Max | 0.8477 |
| 25th %ile | 0.0026 |
| 50th %ile | 0.0050 |
| 75th %ile | 0.0113 |
| 85th %ile | 0.0185 |
| 90th %ile | 0.0275 |
| 95th %ile | 0.0477 |
| 99th %ile | 0.0925 |

**Supplementary Table S4 | Results of susceptibility threshold sensitivity tests.**

| **Threshold (SI)** | **Approx. Percentile** | **Voxels Above Cutoff (%)** |
| --- | --- | --- |
| 0.015 | ~82nd | 18% |
| 0.020 | ~87th | 13% |
| 0.030 | ~91st | 9% |

**Supplementary Table S5 | Key inversion parameters used in the VOXI magnetization vector inversion(MVI) model.**

| **Parameter** | **Setting used in this study** |
| --- | --- |
| Inversion type | 3D voxel-based MVI |
| Grid dimensions | 41 x 43 x 27 |
| Cell size (horizontal) | 250 m × 125 m |
| Vertical extent | Surface to ~6315 m |
| Data uncertainty | 5 nT (uniform absolute) |
| Misfit formulation | Normalized χ² per datum (Eq. 5) |
| Target misfit | $\phi_{d}\approx1$ |
| Final misfit | 0.998 |
| Regularization type | L2 smoothness |
| Gradient weighting | Equal in x, y, z |
| Reference model | None |
| Bounds | None |
| Starting model | Zero magnetization |
| Depth weighting | Applied (Li & Oldenberg-type decay compensation) |
| Trade-off parameter | Auto-fit (VOXI) |
| Iterations | 11 |
