# Supplementary material for: Three dimensional magnetization structure of the Tofua Arc 12 seamount constrained by magnetization vector inversion
Source: Sci Rep. 2026 Apr 3;16:15960. doi: 10.1038/s41598-026-46834-x (PMC13194782; doi:10.1038/s41598-026-46834-x)

**Supplementary Fig. S1 | Stability of RTP magnetic anomaly patterns.**

RTP magnetic anomaly maps (nT) computed using the reference geomagnetic field parameters (I = -42.8°, D = 13.4°) and perturbed values (±10° in inclination and declination), together with the analytic signal amplitude (nT/m). First-order anomaly patterns, including rim-focused maxima, remain stable across perturbations.

Figure S1 was generated using MATLAB R2024b (MathWorks, https://www.mathworks.com/).


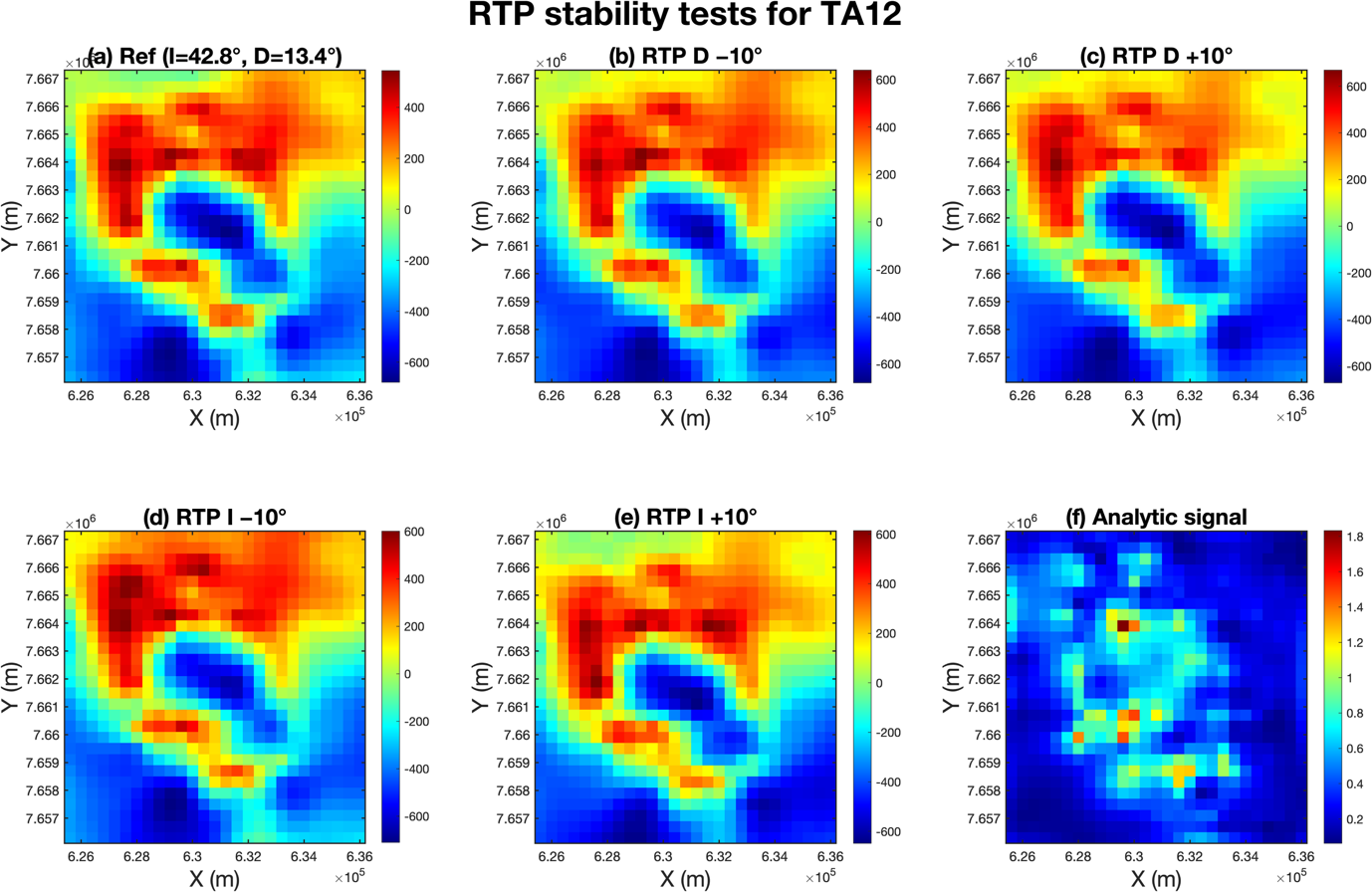


**Supplementary Fig. S2 | Relationship between magnetic anomalies and bathymetric slope at TA12.**

Comparison between magnetic anomaly patterns and bathymetric slope, with a density-based scatter representation. The weak overall correlation indicates that the dominant magnetic anomalies are not primarily controlled by topographic gradients. Bathymetric slope derived from the multibeam grid (degrees).

Figure S2 was generated using MATLAB R2024b (MathWorks, https://www.mathworks.com/).


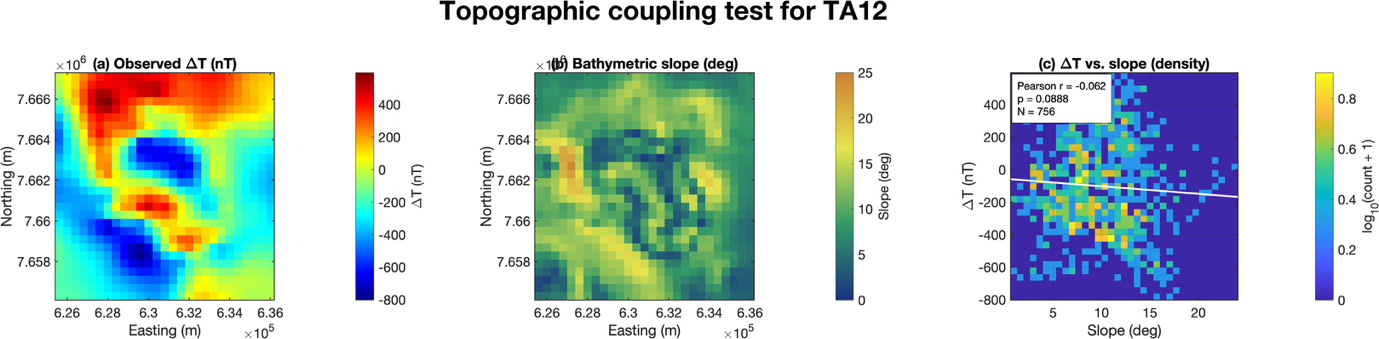


**Supplementary Fig. S3 | Distribution of MVI susceptibility values.**

Histogram of voxel susceptibility values from the MVI model, showing a strongly skewed distribution toward low amplitudes. Vertical dashed lines mark the cutoff thresholds (0.015, 0.020, 0.030 SI) used for sensitivity tests. The inset highlights the low-amplitude range relevant to threshold selection. Susceptibility values are expressed in SI units.

Figure S3 was generated using MATLAB R2024b (MathWorks, https://www.mathworks.com/).


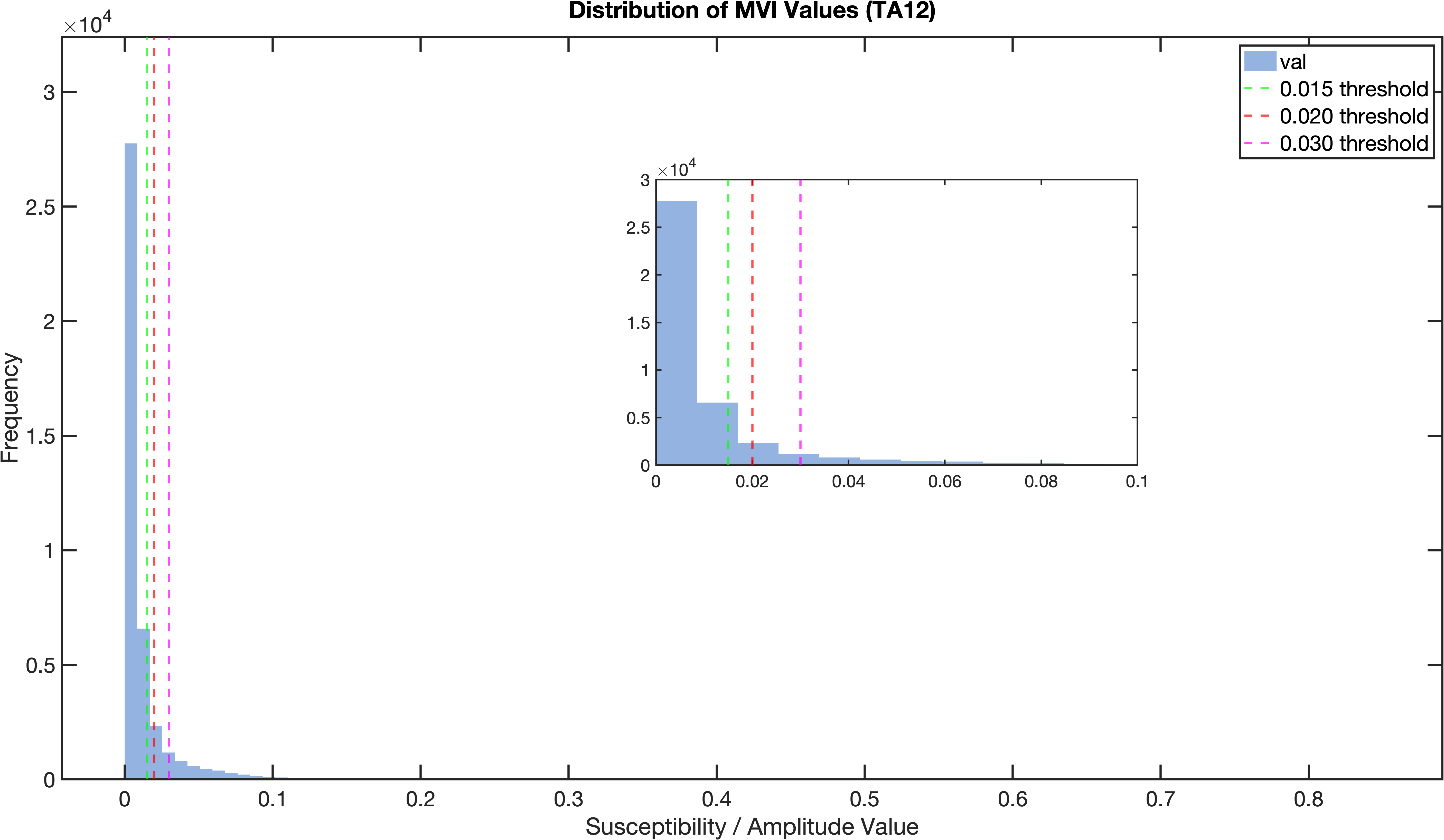


**Supplementary Fig. S4 | Inversion fit diagnostics for the TA12 MVI model.**

Observed, predicted, and residual magnetic anomaly fields used to evaluate the inversion fit. Residuals are small in amplitude and lack coherent spatial patterns, indicating that the recovered model adequately reproduces the first-order anomaly field. Observed, predicted, and residual magnetic anomaly fields (nT) used to evaluate inversion fit based on normalized χ² per datum.

Figure S4 was generated using MATLAB R2024b (MathWorks, https://www.mathworks.com/).


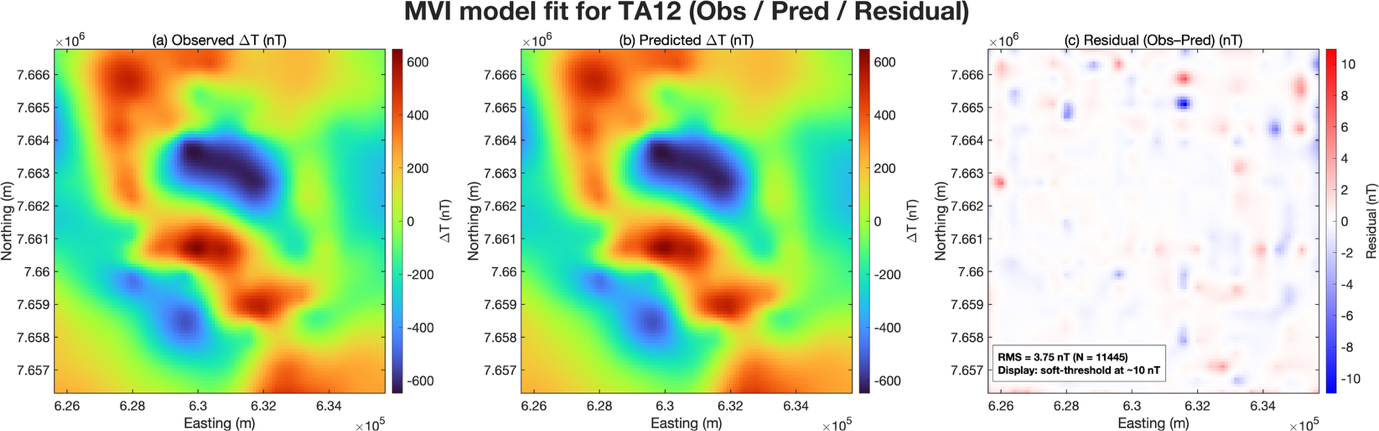


**Supplementary Fig. S5 | Sensitivity of high-susceptibility distributions to cutoff thresholds.**

Spatial distributions of voxels exceeding three susceptibility thresholds (0.015, 0.020, 0.030 SI), shown as 3D point clouds and horizontal slices at representative depths. The persistence of rim-focused high-susceptibility anomalies across thresholds confirms the robustness of the MVI results. Susceptibility values expressed in SI units.

Figure S5 was generated using MATLAB R2024b (MathWorks, https://www.mathworks.com/).


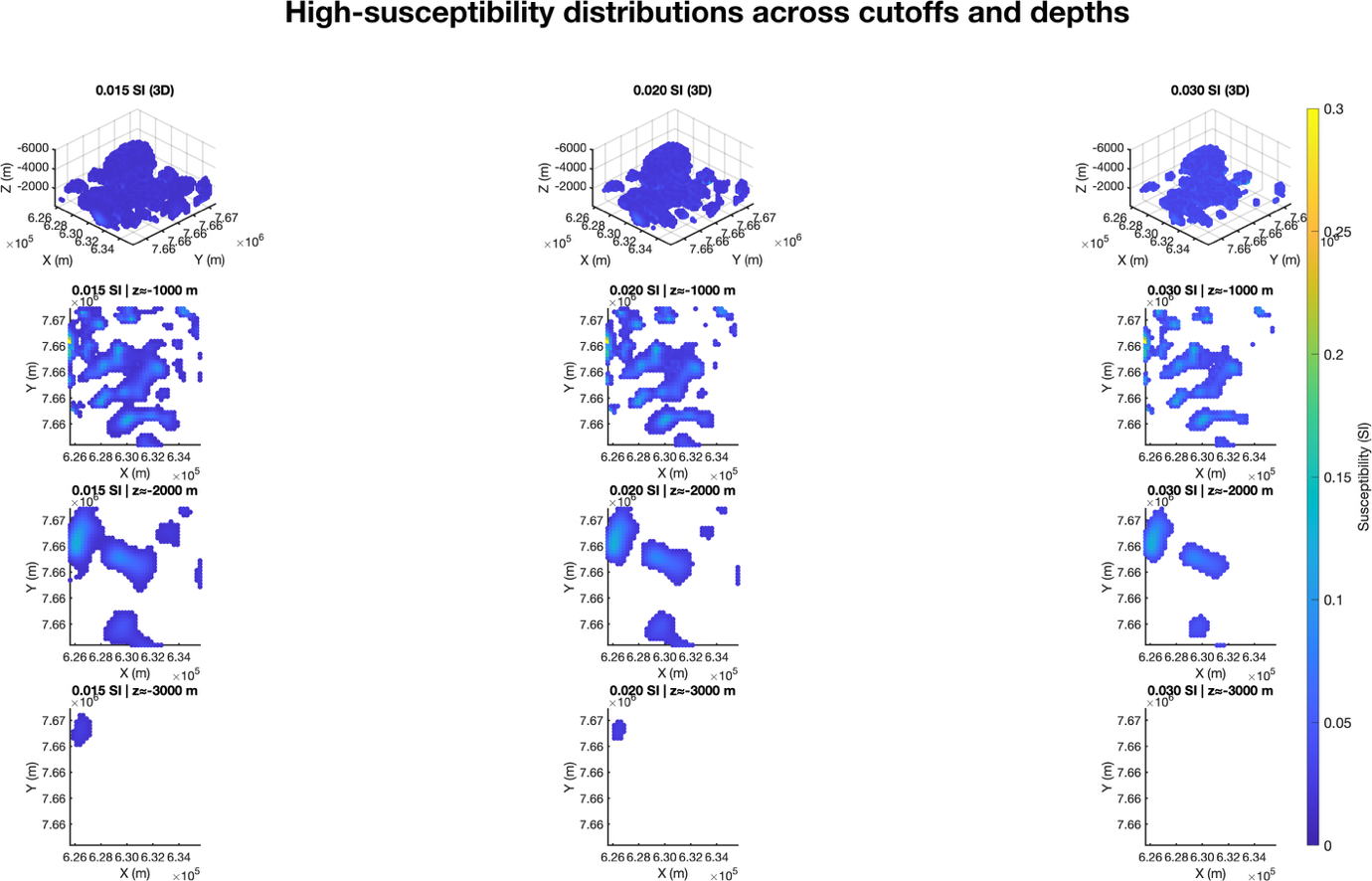

Supplement: Supplementary file 2 — Supplementary Material 2 [file 41598_2026_46834_MOESM2_ESM.docx]
